# Ocular Graft-versus-Host Disease in a Chemotherapy-Based Minor-Mismatch Mouse Model Features Corneal (Lymph-) Angiogenesis

**DOI:** 10.3390/ijms22126191

**Published:** 2021-06-08

**Authors:** Uta Gehlsen, Daniela Stary, Martina Maass, Katarina Riesner, Gwen Musial, Michael E. Stern, Olaf Penack, Philipp Steven

**Affiliations:** 1 Division of Dry-Eye and Ocular GVHD, Department of Ophthalmology, Faculty of Medicine and University Hospital Cologne, University of Cologne, Kerpener Strasse 62, 50924 Cologne, Germany; uta.gehlsen@uk-koeln.de (U.G.); hess.daniela01@gmail.com (D.S.); martina.maass@uk-koeln.de (M.M.); gwen.musial@uk-koeln.de (G.M.); michaelestern4@gmail.com (M.E.S.); 2 Charité—Universitätsmedizin Berlin, Corporate Member of Freie Universität Berlin and Humboldt-Universität zu Berlin, Hematology, Oncology and Tumorimmunology, Augustenburger Platz 1, 13353 Berlin, Germany; katarina.riesner@charite.de (K.R.); olaf.penack@charite.de (O.P.); 3 Berlin Institute of Health at Charité, Universitätsmedizin Berlin, Charitéplatz 1, 10117 Berlin, Germany; 4 ImmunEyez LLC., Irvine, CA 92606, USA; 5 CECAD-Cluster of Excellence in Aging Research at the University of Cologne, University of Cologne, Josef-Stelzmann-Strasse 26, 50931 Cologne, Germany

**Keywords:** ocular graft-versus-host-disease, oGVHD, lymphangiogenesis, chemotherapy, pre-clinical model, blepharitis

## Abstract

Ocular graft-versus-host disease (oGVHD) is a fast progressing, autoimmunological disease following hematopoietic stem cell transplantation, leading to severe inflammation of the eye and destruction of the lacrimal functional unit with consecutive sight-threatening consequences. The therapeutic “window of opportunity” is narrow, and current treatment options are limited and often insufficient. To achieve new insights into the pathogenesis and to develop new therapeutic approaches, clinically relevant models of oGVHD are desirable. In this study, the ocular phenotype was described in a murine, chemotherapy-based, minor-mismatch GVHD model mimicking early-onset chronic oGVHD, with corneal epitheliopathy, inflammation of the lacrimal glands, and blepharitis. Additionally, corneal lymphangiogenesis was observed as part of oGVHD pathogenesis for the first time, thus opening up the investigation of lymphangiogenesis as a potential therapeutic and diagnostic tool.

## 1. Introduction

Ocular graft-versus-host-disease (oGVHD) is a potentially sight-threatening pathology that may affect all tissues of the eye, including the ocular surface, retina, and optic nerve, and it occurs in 40–60% of patients following allogeneic hematopoietic stem cell transplantation (aHSCT) [1,2]. Besides acute forms that occur early, chronic oGVHD is frequent, and onset is months to years after aHSCT. In many patients, chronic oGVHD progresses rapidly and leads to severe ocular surface disease with autoimmunologically-driven destruction of corneal and conjunctival epithelium, and lacrimal gland with attendant tear film deficiency and altered composition. In this process, loss of tissue function, vascularization, and fibrosis result in impaired vision or blindness and ocular discomfort or pain [3]. Despite increasing knowledge of this pathology, there are still significant gaps in our understanding of the underlying immunological and other pathophysiological processes leading to oGVHD, including a potential involvement of lid margins and Meibomian glands in the development of oGVHD [4]. Diagnostic and therapeutic options are insufficient, and preventive approaches are not yet established. This leads to unmet needs for (i) a better understanding of basic pathomechanisms and risk factors leading to the onset of the disease, and (ii) identification of new targets enabling multi-factorial therapies addressing not only inflammation but also fibrosis and pain.

In addition to natural history studies, with collection and analysis of biological samples, basic scientific research is warranted, using clinically relevant GVHD models that allow for translating promising results and targets into the clinic [5]. Animal models of GVHD have been established and provided important insights into pathomechanisms of acute and chronic GVHD, such as the role of dendritic cells, T- and B-cells [6,7,8], T regulatory-cells [9,10], hem- and lymphangiogenesis, etc. [11,12]. However, the majority of GVHD mouse models are based on lethal total body irradiation (TBI) as a conditioning regimen, which is followed by either major histocompatibility complex (MHC)-mismatched or MHC-matched/minor histocompatibility antigen (miHA)-mismatched transplantation of bone marrow and (splenic or lymph node) T cells [13]. This is in contrast to the current standard conditioning treatment in humans that includes cytotoxic drugs (e.g., cyclophosphamide, fludarabine, and busulfan), rather than TBI [14]. Furthermore, the absolute majority of aHSCT patients are transplanted HLA-matched or only partially mismatched [15,16]. Since the degree of the mismatch, as well as TBI, influences the severity of experimental GVHD, and probably also of oGVHD, suitable mouse models for ocular GVHD are desirable.

Regarding the eye, several pre-clinical models are established to specifically investigate ocular manifestations of GVHD [17,18,19,20], as recently reviewed in [4]. In these models, several phenotypes similar to either acute or chronic ocular GVHD in humans are described and investigated further, including inflammation and fibrosis of the cornea, conjunctiva, lid marging, and lacrimal glands [18,19,21,22,23,24]. Some of these models use GFP-overexpressing cells [21].

However, the stratification of acute vs. chronic ocular GVHD in these models however requires further clarification, as in humans, acute ocular GVHD demonstrates specific different clinical features than chronic ocular GVHD. Instead, frequently used animal models of acute systemic GVHD may not present an acute ocular phenotype with e.g., pseudomembranes, epithelial sloughing, and chemosis. Furthermore, although using MHC-matched, miHA-mismatched donors, established models for ocular GVHD are all based on TBI. In this context, it is known that TBI has dose-dependent effects on the severity of systemic GVHD, but also on ocular tissues, with more severe involvement of lid margins and corneal ulceration [21].

To better relate to the current situation of allogeneic, hematopoietic stem cell transplantation in humans, we chose a recently described, minor-mismatch mouse model that utilizes chemotherapy instead of TBI as a conditioning regimen [11,25]. Using this model, the first aim was to describe and analyze the ocular phenotype in detail while using established grading systems [21] or introducing modifications where necessary. Furthermore, as in the same model, lymphangiogenesis and hemangiogenesis were described as a key pathomechanism of systemic GVHD, in particular within the intestine [11,26], and the inhibition of lymphangiogenesis ameliorated GVHD and improved survival in mice [11], we wanted to further test the hypothesis that lymphangiogenesis would also occur in the eye, namely within the normally avascular cornea. As corneal lymphangiogenesis is thought to contribute to the development of ocular surface disease, in particular dry-eye disease, as recently reviewed in [27], it could serve as a new therapeutic target for ocular GVHD [28,29].

## 2. Results

### 2.1. Systemic GVHD Manifestations

**Figure 1:**
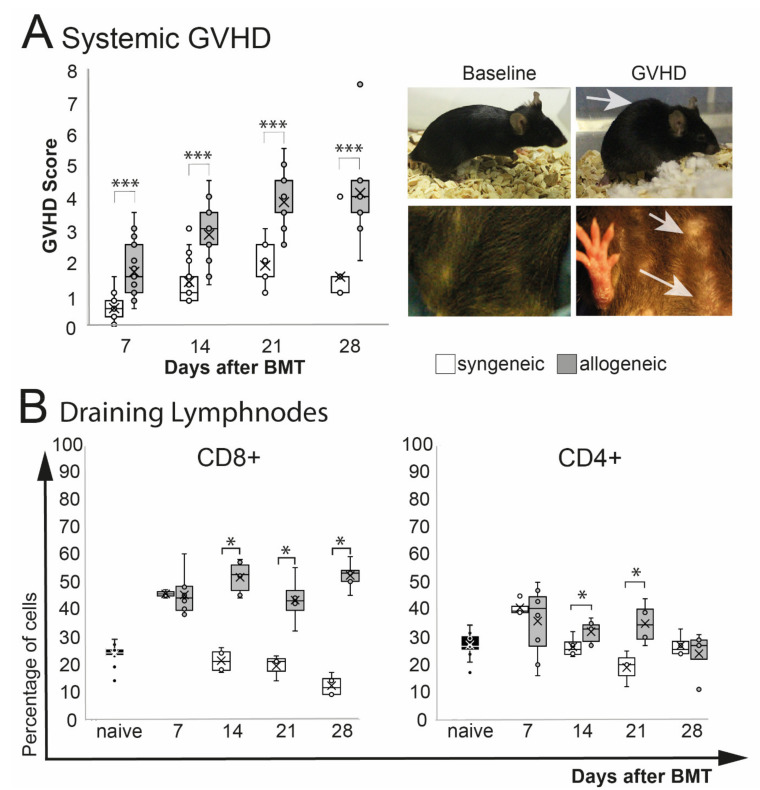
Systemic phenotype after chemotherapy and either syngeneic (B6-B6) or allogeneic (129s/B6) BMT, respectively. GVHD score contained weight loss, fur and skin condition, posture, and overall activity. (**A**) Allogeneic transplanted mice demonstrated a significant GVHD burden. Exemplary macroscopic images showing typical hunched back (upper right photo) and scaled skin of allogeneic GVHD mice (lower right photo; see arrows) compared to baseline. (**B**) Flow cytometry analysis of eye-draining lymph nodes. Compared to syngeneic controls allogeneic mice showed a significantly higher percentage of CD8+ T cells and CD4+ T cells from day 14 after BMT. (* *p* ≤ 0.05, *** *p* < 0.0001).

A significant increase of phenotypical signs of systemic GVHD (weight loss, poor posture, reduced activity, abnormal fur and skin condition) was observed in allogeneic recipients after BMT (*p* = 0.0001) (Figure 1A). The overall GVHD score in allogeneic mice peaked at d21 after BMT and remained on that level until the end of the observation at d28. Allogeneic GVHD mice showed a significant weight loss after chemotherapy treatment, which improved 1–2 weeks after BMT. Two weeks after, BMT allogeneic transplanted mice showed a general deterioration of the overall condition, with reduced activity, hunched posture, abnormal, dull fur, and flaked skin (Figure 1A). No differences in the overall survival between syngeneic and allogeneic were observed (log-rank test: *p* = 0.4) (Supplemental Scheme S1). Analysis of the eye-draining lymph nodes revealed a significant increase of CD8+ T cells (*p* = 0.04) and by trend in CD4+ T cells (*p* = 0.09) in allogeneic mice compared to naïve mice, but there was no change in syngeneic mice (*p* = 0.1, CD8+ and CD4+ each). Compared to syngeneic control mice, CD8+ T cells in allogeneic transplanted mice were increased significantly at days 14 and 21 and remained elevated until the end of the observation period at day 28. The number of CD4+ T cells was increased at days 14 and 21 in allogeneic mice but returned to baseline level in syngeneic and allogeneic mice at day 28 after BMT (Figure 1B).

### 2.2. Ocular GVHD Manifestations

**Figure 2:**
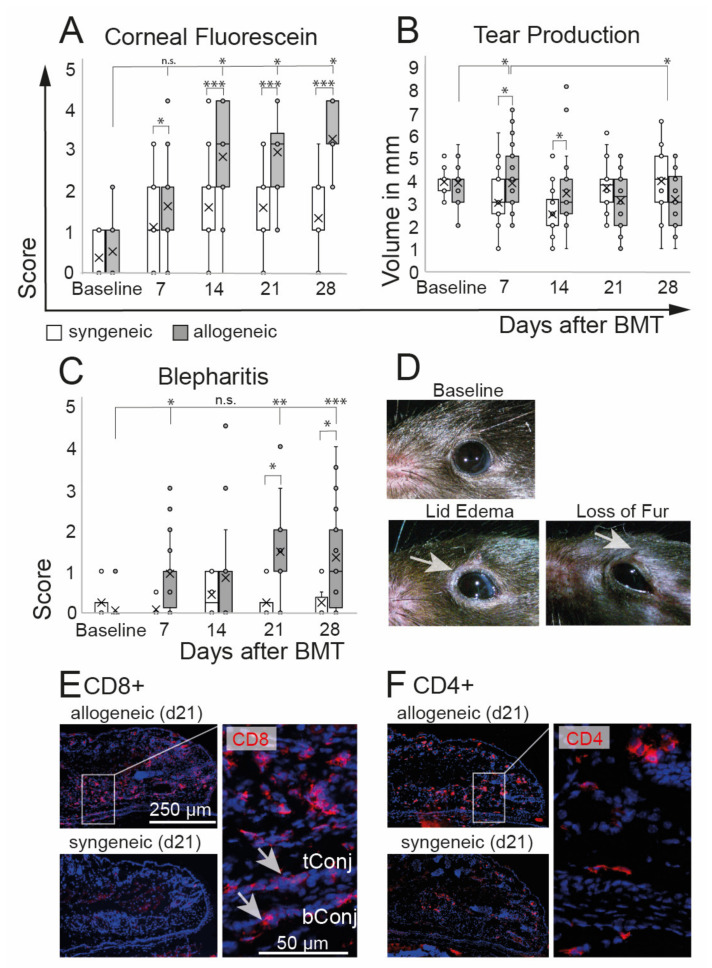
Ocular phenotype after chemotherapy and either syngeneic (B6-B6) or allogeneic (129-B6) BMT, respectively. (**A**) Corneal epitheliopathy increased in allogeneic mice 2 weeks after BMT compared to baseline and was significantly worse compared to syngeneic controls at each time point after BMT. Syngeneic mice developed no epitheliopathy after BMT. (**B**) Tear production remained almost constant in both groups until the end of the observation period at d28 after BMT. A minor increase of tears was observed in allogeneic mice compared to baseline 7 days after BMT and compared to syngeneic controls at days 7 and 14 after BMT. (**C**,**D**) Blepharitis score (including lid edema and loss of fur, see (**D**) exemplary photos) was significantly increased in allogeneic mice compared to syngeneic mice three weeks after BMT. Blepharitis score remained at the baseline level in syngeneic mice. (**E**,**F**) Immunohistochemistry of frozen tissue sections of eyelids at day 21 after BMT. Allogeneic mice present distinct inflammatory infiltration of CD8+ (**E**) and CD4+ (**F**) T cells in the lids and conjunctival tissues (see inlays) (tConj/bConj = tarsal/bulbar conjunctiva; * *p* ≤ 0.05, ** *p* < 0.001, *** *p* < 0.0001).

Clinical assessment of oGVHD was performed using corneal fluorescein staining scores, tear production, and degree of blepharitis. Allogeneic recipients developed a distinct ocular GVHD phenotype with a significant increase of corneal staining that was observed from d14 on (*p* = 0.0001) and that was significantly worse compared to syngeneic controls over the entire duration of the experiment. In syngeneic recipients, corneal staining was slightly increased at d7 (*p* = 0.003) without a further increase (Figure 2A). A significant increase in tear production was observed in allogeneic mice at d7 compared to baseline as well as to syngeneic controls at d7 and d14. At d28, the tear production in allogeneic mice was reduced compared to d7 (Figure 2B). Inflammation of the lids was measured using a novel blepharitis score (Figure 2C,D) and histological investigations of the lids (Figure 2E,F). Allogeneic recipients developed severe blepharitis, including swelling of the lids and loss of fur from d7, with a peak at d21 and d28 after BMT compared to baseline (*p* = 0.0001). In syngeneic controls, no changes of lid margins in comparison to baseline were observed (*p* = 0.9). Immunohistological investigations revealed distinct infiltrations of CD8+ and CD4+ T cells in the lids, including the tarsal and bulbar conjunctiva of allogeneic mice. Representative images of allogeneic and syngeneic recipients are shown in Figure 2E,F.

### 2.3. Inflammation of the Lacrimal Gland

**Figure 3:**
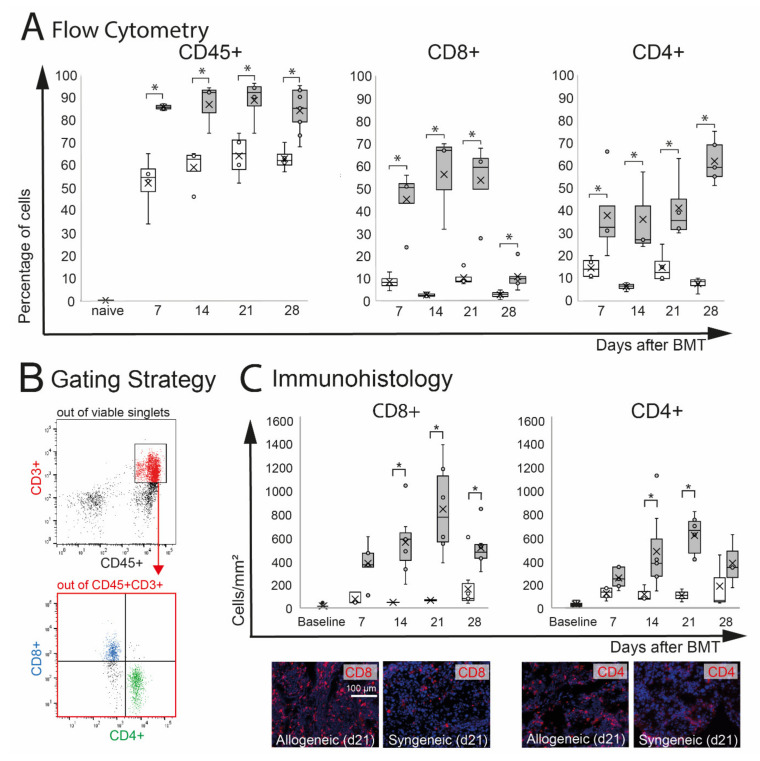
Effect of chemotherapy conditioning followed by either syngeneic (B6-B6) or allogeneic (129s-B6) BMT on extra-orbital lacrimal glands. (**A**) Flow cytometry analysis of ELG tissue. A significant infiltration with CD45+ leucocytes was observed in both groups compared to naïve mice. Allogeneic mice developed more pronounced CD45+ infiltration compared to syngeneic control mice. In allogeneic mice, the numbers of CD8+ and CD4+ cells were significantly increased compared to syngeneic mice. (**B**) Gating strategy. After excluding dead cells and doublets, viable cells were gated on CD45+CD3+ cells, and the number of CD8+ and CD4+ T cells was calculated. (**C**) Immunohistochemistry of frozen sections. The number of positive cells was counted manually and calculated per mm^2^. The results confirmed significant infiltration with CD8+ and CD4+ T cells in ELG tissue of allogeneic mice. In allogeneic mice, a higher number of CD8+ and CD4+ cells per mm^2^ was found at d14 and d21 after BMT (* *p* ≤ 0.05).

Extra-orbital lacrimal glands (ELG) are target organs in oGVHD and were significantly affected in this model. In ELG, inflammatory infiltration with CD8+ and CD4+ T cells was observed. Flow cytometry analysis demonstrated a significant increase of CD45+ leucocytes in allogeneic (*p* = 0.05) and by trend in syngeneic (*p* = 0.06) ELG compared to naïve controls, which showed almost no CD45+ infiltration. The percentage of CD45+ cells was significantly higher in allogeneic recipients compared to syngeneic controls at d7–28 (Figure 3A). Out of the CD45+ cells, the number of CD3+CD8+ and CD3+CD4+ T cells in ELG was calculated (Figure 3B). As a result of the very low number of CD45+ CD3+ cells in naïve ELG, the percentage of CD8+ and CD4+ was only calculated for allogeneic and syngeneic mice d7-d28. In allogeneic ELG, significantly higher percentages of CD8+ and CD4+ T cells were observed compared to syngeneic controls at the time points investigated (d7–d28). The immunohistochemistry of ELG sections confirmed severe CD8+ and CD4+ T cell infiltration in the tissues, reflecting the results of the flow cytometry with an increased number in allogeneic recipients compared to syngeneic at d14, 21, and 28. At d7, the numbers were increased by trend in allogeneic mice (CD8+: *p* = 0.06; CD4+: *p* = 0.07). Representative images of allogeneic and syngeneic ELG sections are presented in Figure 3C.

### 2.4. Corneal Hem- and Lymphangiogenesis

**Figure 4:**
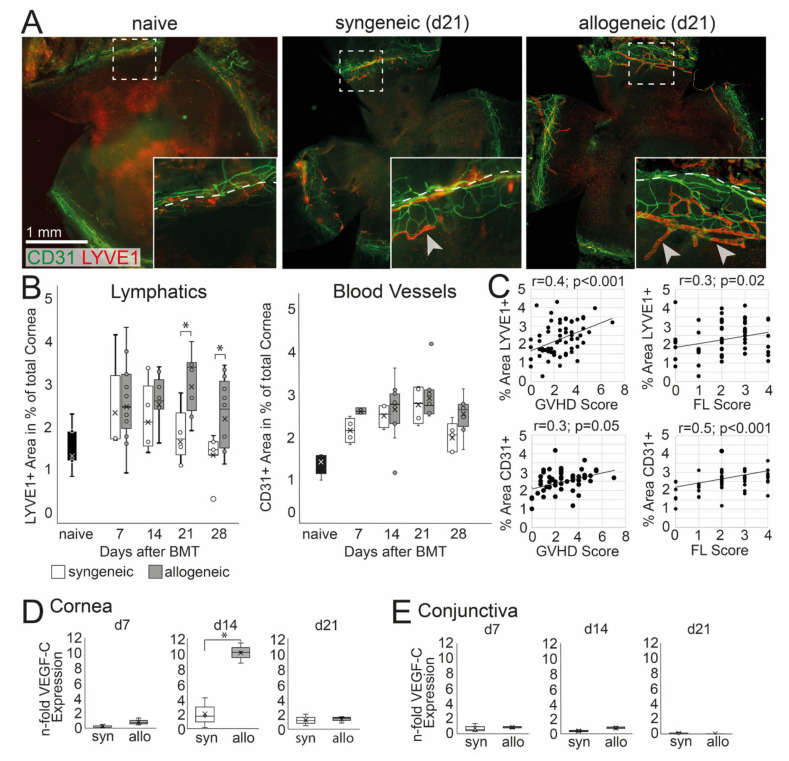
Corneal hem- and lymphangiogenesis in oGVHD. (**A**) Exemplary corneal flat-mount samples of naïve, syngeneic, and allogeneic transplanted mice 21 days after BMT (CD31 = green, Lyve1 = red; bar = 1 mm). (**B**) Average area covered by Lyve1+ lymphatics and CD31+ blood vessels in percent of the total cornea. 21–28 days after BMT, a significant increase of lymphatics was observed in allogeneic mice compared to syngeneic control. No differences in corneal blood vessels were observed. (**C**) Corneal hem- and lymphangiogenesis was correlated to the systemic GVHD score and the corneal fluorescein score (FL). (**D**,**E**) VEGF-C expression in the cornea (**D**) and conjunctiva (**E**) normalized to naïve controls. In the cornea, but not in the conjunctiva, the VEGF-C expression was increased in allogeneic mice compared to syngeneic control 14 days after BMT (* *p* ≤ 0.05).

Corneal hem- and lymphangiogenesis was analyzed and then correlated with systemic and ocular GVHD. An ingrowth of LYVE-1+ lymph vessels into the normally avascular cornea was observed in allogeneic and to a lesser extend in syngeneic mice (Figure 4A). At d21 and d28 after BMT, lymphangiogenesis was significantly higher in allogeneic mice, and a larger area of the cornea was covered with LYVE-1+ lymphatics compared to syngeneic mice (d21 *p* = 0.05, d28 *p* = 0.04) (Figure 4B). In addition, CD31+ blood vessels were observed in the cornea; however, the ingrowth of blood vessels was not significantly increased in allogeneic recipients compared to syngeneic control or naïve mice (Figure 4B). The increase of lymph vessels was accompanied by an up-regulation of VEGF-C in the cornea at d14 in allogeneic but not in syngeneic mice (Figure 4D). In addition, there was no change in the expression of VEGF-C in the conjunctiva or at later time points after BMT in both groups compared to naïve mice (Figure 4E). To analyze a potential correlation between oGVHD severity and corneal hem- and lymphangiogenesis, a Pearson correlation was executed. Lymphatics, as well as blood vessels, were positively correlated with systemic GVHD score (lymphatics: *p* = 0.0001, blood vessels: *p* = 0.05) and corneal epitheliopathy (FL score) (lymphatics: *p* = 0.02, blood vessels: *p* = 0.0001) with medium effect size (r = 0.3–0.5).

## 3. Discussion

In this study, we describe for the first time ocular manifestations in a minor-mismatch GVHD mouse model, using chemotherapy as conditioning regimen [11,25]. The main features of this model are (i) close accordance with human conditioning and transplantation procedures and (ii) its reliable development of moderate-severe GVHD symptoms, but without hyper-acute, lethal progression of the disease, as often observed in other GVHD models. Therefore, this model enables the investigation of ocular GVHD in an experimental setting with overall acceptable survival rates. Previously, a zenith of systemic GVHD was reported around day 15–20 after transplantation [25], which was comparable to our study. Regarding ocular GVHD, we found a significant onset of disease, starting at d14 after BMT, with increasing corneal epitheliopathy and severe blepharitis caused by lymphocyte infiltration in allogeneic recipients correlating with an increased number of CD8+ T cells in the cervical draining lymph nodes.

As described above, cases that demonstrate severe onset of inflammation of cornea, conjunctiva, and lid margin that rapidly progress into tissue destruction and fibrosis with consecutive loss of visual function are the most relevant forms in the clinic [3,30]. Here, early diagnosis of these forms of ocular GVHD, which are mostly classified as chronic ocular GVHD, present with a rather narrow window of opportunity to avoid irreversible damage of the eye [31]. Currently, it is not well understood which pathophysiological mechanisms are taking place in this early onset, rapidly progressing form of ocular GVHD, and except for topical corticosteroids and calcineurin antagonists, no well-established therapeutic options are available [30]. Therefore, a suitable animal model that would enable investigating early immunological processes would help identify potential targets for more specific drugs.

The model described here shares typical features of the described early-onset chronic ocular GVHD phenotype. Corneal staining as well as blepharitis develops early after experimental BMT and remains at a high level until the end of the study, indicating ongoing inflammation at the ocular surface. This is in contrast to previously established ocular GVHD animal models, which demonstrated blepharitis and immune cell infiltration in the lids at 3–4 weeks after BMT: the earliest and peaking 6–7 weeks after BMT [19,21]. The severity of the corneal and lid infiltrations in those studies was also correlated to the dose of irradiation conditioning [21], whereas the severity of blepharitis in our model was not accelerated by chemotherapy treatment. However, more detailed experiments with different chemotherapy dosages are needed to fully answer that question. Regarding the eye and its adnexa, we observed an increase in tear production at d7, which was probably caused by the initial irritation of the ocular surface and can lead to increased reflex tearing. We found a strong CD8+ T cell infiltration in the lacrimal glands starting at d7 until d21 after BMT, which decreased at d28, but a constant increase of CD4+ T cells over time suggesting chronic inflammation of the lacrimal glands and immune suppression of the lacrimal function, which could explain the decrease of tears at d28. This correlates to a distinct CD8+ T cell-driven systemic GVHD peak at d14, which was followed by a CD4+ T cell-driven phase at later time points, as well as scleroderma-like chronic GVHD symptoms, as reported in [25]. Comparable inflammatory cellular infiltrates within the lacrimal glands were also described in an irradiation-based, minor mismatch GVHD model using histological methods, together with apoptosis and destruction of the acini starting from week 2–4 after BMT and peaking at 6 weeks after BMT [17]. It is important to note that different to other target organs of GVHD such as skin, our findings in the eye do not resemble acute ocular GVHD, which is characterized by chemosis and pseudomembranes as well as a rather severe onset of chronic ocular GVHD. Chronic GVHD involves fibrotic and autoimmune tissue alterations, leading to chronic inflammation, but it is only poorly understood. Corneal inflammation and keratinization were recently mentioned in a chronic TBI-GVHD model [22], but the ocular GVHD phenotype was not investigated further. In this study, we did not hover to let the model continue until vast fibrotic changes might be visible. Therefore, more detailed studies are needed to better understand the underlying pathomechanisms leading to acute and chronic oGVHD. Using this model will open up the possibility to investigate the development of oGVHD from acute to chronic forms that are clinically more frequent and of particular interest.

Addressing the second aim of the study, we demonstrated for the first time that corneal lymphangiogenesis takes place in experimental oGVHD. It has been shown previously that hem- and lymphangiogenesis are features of and initiate acute systemic GVHD [11,26]. Angiogenesis is present in very early stages, preceding CD8+ and CD4+ T cell infiltration into specific target tissues of GVHD such as the intestine and liver. Furthermore, previous studies could demonstrate that the experimental inhibition of hemangiogenesis protected against the infiltration of inflammatory leukocytes in the skin and intestine [12], and it showed beneficial effects on the survival and severity of experimental GVHD [26]. The same group found that endothelial activation by adhesion molecules or VEGF-A did not play a role [26], whereas, in addition, lymphangiogenesis in the intestinal tract and lymph nodes could be specifically targeted using VEGF-R-3 antibodies and ameliorated GVHD [11]. In contrast to the intestine or skin, the cornea possesses an angiogenic privilege and is, confined by the limbus, entirely avascular to maintain transparency under physiologically and healthy conditions (reviewed in [32]). In inflammatory diseases, such as dry-eye [28] and stromal keratitis [33], after corneal injury, or after surgical procedures [34], pathological neovascularization can occur, leading to a loss of the angiogenic and also the immune privilege of the eye with potentially devastating consequences [35]. For these reasons, intensive research has been done to better understand in particular lymphangiogenesis in ocular diseases and to develop new therapeutic interventions (reviewed in [27,36]). In a murine dry-eye model, the systemic blocking of VEGF-C reduced pathological lymphatics in the cornea, which was correlated with reduced clinical signs of dry-eye and less inflammation, e.g., decreased numbers of cellular infiltrates in the cornea and decreased levels of pro-inflammatory cytokines in the conjunctiva [37]. Our data now indicate that the cornea is also a target tissue in GVHD-related lymphangiogenesis correlating with systemic GVHD severity. The question remains of whether lymphangiogenesis is only induced as part of GVHD pathomechanisms or is also triggered by a conditioning regimen. Regarding the gut, it was shown that early angiogenesis was not induced by chemotherapy or by syngeneic transplantation alone [26]. In our study, corneal lymphatics seemed to be slightly increased in syngeneic controls (however, this was statistically non-significant). This implicates a potential role of chemotherapy, e.g., by endothelial damage, as discussed in [38]. The significant increase of corneal lymphatics in the allogeneic group was preceded with an upregulation of VEGF-C, which is an activator of lymphangiogenesis. It is known that different mice strains show differences in the number of physiological lymphatics in the limbal region due to a genetic heterogeneity [39], suggesting also different responsiveness to angiogenic triggers. In C57/Bl6 mice (used as recipients in this study), more pronounced response to VEGF-C induced corneal neovascularization was reported compared to other mouse strains [40]. Therefore, the ingrowth of lymphatics at early time points could be influenced by chemotherapy treatment, whereas the later significant increase of corneal lymphatics in allogeneic recipients is directly caused by GVHD. Overall, despite these findings, the exact pathophysiological role of lymphangiogenesis in oGVHD needs to be elucidated. We here hypothesize that VEGF-C secretion follows a certain level of inflammation and tissue damage; then, it induces lymphangiogenesis, which further triggers the immune response. Hereby, lymphangiogenesis could depict a milestone in the chronification of disease. Therefore, anti-lymphangiogenic therapies could result in less pronounced disease progression. Furthermore, a potential influence of chemotherapy on ocular GVHD pathogenesis, as demonstrated for GVHD of oral mucosa, lung, and intestine [38], needs to be investigated.

In conclusion, this minor-mismatch mouse model using chemotherapy as a conditioning regimen opens up the possibility to investigate in depth the development of early-onset chronic forms of oGVHD after allogeneic stem cell transplantation, with high potential of translating results into the human situation. Furthermore, for the first time, corneal lymphangiogenesis is described as a potential pathomechanism in oGVHD and as a promising therapeutic target and an in vivo biomarker [41,42].

## 4. Materials and Methods

### 4.1. Animal Experiments

Female mice, 12 weeks old, C57BL/6 (H2k^b^) (recipients) and 129S2/SvPasCrl (H2k^b^) (donors), were purchased from Charles River Laboratories (Sulzfeld, Germany). Mice had access to food and water ad libitum. All experiments were approved by the local Ethics Committee for Animal Experiments (State of North-Rhine Westfalia, Germany) and complied with the ethical principles for the use of animals in ophthalmic research stated by ARVO. All animals were monitored daily to ensure their well-being. To avoid excessive burden and major pain, a stop criterion was defined. Animals that reached the criterion were immediately sacrificed without pain.

#### 4.1.1. Conditioning Regimen and Bone Marrow Transplantation (BMT)

GVHD was induced as described previously in detail [25]. To eliminate the immune system, the recipient mice received IP injections with busulfan (Sigma-Aldrich (St. Louis, MO, USA); 20 mg/kg/day for 4 days), followed by cyclophosphamide monohydrate (Sigma-Aldrich; 100 mg/kg/day for 2 days). After two days of rest, the recipients were injected IV with 1.5 × 10^7^ bone marrow cells (BMC) and 2 × 10^6^ splenic T cells from 129S2/SvPasCrl (allogeneic) or C57BL/6 (syngeneic control) donor mice. BMC were flushed from tibia and femur, erythrocytes were lysed, and the BMC suspension was washed two times in PBS/2% FCS/1 mM EDTA and filtered to obtain a single-cell suspension (70 µm cell strainer, BD-Bioscience). A T cell suspension was prepared using a magnetic-sorting pan T cell isolation kit (Miltenyi Biotec, Bergisch Gladbach, Germany) according to the manufacturer’s instructions. After the BMT mice were observed for 4 consecutive weeks (28 days). The recipients were examined twice a week for clinical parameters of systemic GVHD including weight(-loss), posture, activity, skin, and fur on a scale from 0 to 2 for each parameter. The severity of systemic GVHD was assessed by summation of the parameters. An overall score of 6 was defined as the stop criterion. At the end of the experiments, flow cytometry analysis of blood samples using the chimerism markers Ly9.1 and H2ƙb were performed to ensure successful allogeneic transplantation and engraftment.

#### 4.1.2. Ocular GVHD

To investigate the ocular GVHD phenotype, mice were screened at baseline and days 7, 14, 21, and 28 after BMT. After a careful macroscopic examination of the eyes, images of the lids were taken to analyze the severity of blepharitis (lid swelling). For this, a new scoring system was developed: swelling of the lids and loss of fur around the eyes were scored from 0 to 2, and the final blepharitis score was assessed by summation of the parameters (see Figure 1F and Supplemental Scheme S2). Tear volume was measured using phenol red threads (Zone Quick Tread, Oasis Medical, San Dimas, CA, USA). A thread was placed into the lateral cantus of the eye for 30 s, and the amount of tear fluid was recorded in mm. Corneal damage was detected by fluorescein staining. Fluoresceine (5 µL of 5% solution in NaCl) was applied topically and carefully wiped off after 30 s without touching the cornea. Images were taken under a blue light source and analyzed using a modified grading scheme with severities ranging from 0 to 5 as described previously [43].

On days 7, 14, 21, and 28 after BMT, allogeneic and syngeneic control recipient mice were sacrificed and cornea, conjunctiva, as well as the lids, extra-orbital lacrimal glands, and draining lymph nodes were excised. Tissue samples of naïve mice were used as a control.

#### 4.1.3. Histology

Sections (8 µm) of fresh frozen lacrimal glands and whole eyes were acetone-fixed and incubated with a final concentration of 1:150 with rat anti-mouse-CD4 (BD Bioscience) and rat anti-mouse-CD8 (Invitrogen) antibodies (AB) 1 h at room temperature. An Alexa-555 conjugated goat anti-rat secondary AB was incubated for 1 h at room temperature in the dark (Invitrogen). Nuclei were stained with Hoechst 1:20,000 (Thermo Scientific, Waltham, MA, USA). For each sample, a negative staining control (secondary AB only) was carried out. Images were taken with an Olympus fluorescence microscope equipped with a BX53 camera. To calculate the number of CD4+ and CD8+ T cells in lacrimal glands, the open-source software ImageJ (v1.48) containing the “cell counter” plugin was used. From each lacrimal gland section, the number of positive cells was counted manually in three representative positions of the gland (area 1376 × 1038 µm each) and averaged. Then, the number of CD4+ and CD8+ cells was depicted in cells/mm^2^.

#### 4.1.4. Flow Cytometry

Excised lacrimal glands were carefully chopped into pieces and digested with 2 mg/mL liberase (Roche, research grade 05401020001) in Krebs-Ringer buffer (ZenBio, Durham, NC, USA). Tissue samples were incubated for 30 min at 37 °C on a shaker. After being fully digested, the suspension was centrifuged (400 G, 5 min), the supernatant was removed, and the pellet was re-suspended in FACS buffer (0.2% HEPES (1M in 0.85 NaCl) and 2% fetal bovine serum (Gibco, Life Technologies, Schwerte, Germany) in Dulbeccos PBS (Sigma Aldrich, Darmstadt, Germany) and filtered through a 40 µm strainer. Single-cell suspensions of lymph nodes were prepared by meshing the tissue through a 40 µl cell strainer. After washing with complete medium (1xRPMI 1640 without L-Glutamine (Gibco, Life Technologies, Schwerte, Germany), 10% fetal bovine serum, 2% penicillin/streptomycin), and blood cells were lysed using Hybrid-Max™ lysing buffer (Sigma) for 1 min on ice. Lysis was stopped by adding complete medium (1:10). After spinning down (10 min, 300 G, 4 °C), the supernatant was removed, and the pellet was re-suspended in FACS buffer. Erythrocytes were lysed, and after washing, the pellet was resuspended in FACS buffer. Cells were blocked with anti-mouse CD16/CD32 FC-block (BD Pharmingen, clone 2.4G2). After 10 min incubation on ice, samples were incubated with CD45, CD3, CD4, and CD8 AB according to the manufacturer’s instructions for 30 min at 4 °C and protected from light (detailed information of antibodies used are listed in the Supplemental Scheme S3). After incubation, cells were re-suspended in serum-free PBS and incubated with eBioscience™ fixable viability dye eFluor™ 780 (Invitrogen) for 20 min in the dark. Then, cells were washed and fixed with BD™ Stabilizing fixative (BD Bioscience, Heidelberg, Germany) 1:3 in aqua dest. Measurements were executed the next day using a BD FACSCanto™II cell analyzer (BD Biosciences). Analysis was done using FlowJo software (FlowJo LLC, OR, USA). After excluding dead cells, doublets, and CD45-negative (non-leucocytes), data were analyzed using FMO controls for each antibody, and the number of CD3+CD4+ and CD3+CD8+ T cells was calculated as presented in Figure 2B.

#### 4.1.5. Real-Time RT-PCR

The cornea and conjunctiva of two eyes/sample were excised, chopped into small pieces, and snap frozen in RLT buffer (added with 10 µL/mL β-mercaptoethanol). Samples were thawed on ice and sonicated 3 × 20 s. Samples were centrifuged (3 min at 10,000 G). Then, RNA was isolated from the supernatant using the Qiagen RNeasy Plus Mini kit with a genomic DNA eliminator spin column according to the manufacturer´s instructions. RNA was diluted in RNase-free water, and the concentration, as well as the purity, were determined using a spectrophotometer device (NanoDrop, Thermo Fisher, Oberhausen, Germany). cDNA was synthesized by reverse transcription using a Revert Aid First-Stand cDNA Synthesis Kit (Thermo Scientific, Bonn, Germany) according to the manufacturer´s instruction. The obtained cDNA was used for a SYBR Green-based quantitative real-time PCR. PCR reactions were performed in a 20 µL volume using 20 ng cDNA and 0.75 µM of each forward and reverse primer (Eurofins Genomics) and SsoFast EvaGreen Supermix (Bio-Rad, Bonn, Germany). Thermal cycling was performed at 95 °C for 2 min followed by 45 cycles of 95 °C for 5 s and 60 °C for 30 s in a CFX96 Touch Real-Time PCR Detection System (Bio-Rad, Bonn, Germany). Real-time PCR results of VEGF-C were analyzed by the comparative threshold method using HPRT (hypoxanthine-guanine phosphoribosyl-transferase) as an endogenous reference gene. All PCRs were executed as triplicate, and a non-template control (water) was included to exclude contamination. The relative mRNA level of naïve mice was used as control, and allogeneic and syngeneic mice were normalized to control. Primer sequences, product size, and annealing temperatures are listed in Table 1.

#### 4.1.6. Semi-Automatic Lymph Vessel Segmentation of Corneal Flat Mounts

Corneal flat-mount tissue was fixed in acetone, blocked with 2% bovine serum albumin in PBS, and incubated (1:100) with a rabbit-anti-mouse-LYVE-1 (Abcam, Cambridge, UK) and a FITC labeled anti-CD31 antibody (BD Pharmingen, San Jose, CA, USA) 4 °C overnight. The next day, corneas were washed, and LYVE-1 was detected with a goat anti-rabbit Cy3-conjugated secondary antibody (Jackson ImmunoResearch, Hamburg, Germany). Whole-mount RGB images were acquired with an Olympus (BX53) fluorescence microscope. Afterward, a custom program (MATLAB; The MathWorks, Inc., Natick, MA, USA) was used to segment the lymphatic vessels from the RGB images. The limbal edge and the inner border of the region of the cornea were manually selected by an experienced user. The limbal edge border was used to calculate the total cornea area for later density measurements. For lymphatic and blood vessel segmentation, either the red or the green channel was isolated; then contrast-enhanced with a diffusion filter. Median filtering (15 × 15 kernel) and background subtraction with a tophat filter (σ = 45) were applied before a Frangi filter (20 and 25 pixels) for vessel enhancement [44]. Then, Otsu’s method was used to threshold the image to convert to binary [45]. Any erroneous segmentation outside the limbal edge or in the central portion of the cornea due to staining artifacts was removed based on the manual masks, and small objects of less than 50 pixels were removed. Then, the binary segmentations were manually corrected in GIMP, a free open-source image editing software. The total number of vessel pixels in the binary segmentation was found in MATLAB and used for later density evaluation. A custom program built in Python was used to compute the total number of lymphatic pixels in the corrected binary segmentations. The lymphatic or blood vessel density was calculated using the number of pixels in the corrected binary segmentation over the number of pixels (1.3 µm/pixel) in the cornea mask created by the user from the MATLAB program.

#### 4.1.7. Data Analysis and Statistics

Experiments were performed at least twice with n = 3–5 mice (6–10 eyes) per group. To avoid bias, each final image analysis (blepharitis, fluorescein, lymphatic and blood vessels, lacrimal gland infiltration) was performed in a blinded manner by two independent, experienced observers.

All data were checked for their Gaussian distribution using the Kolmogorov–Smirnov test. For statistical analysis, the non-parametric Friedmann Test (with Bonferroni corrected post hoc for multiple testing) and the Mann–Whitney-U Test were performed to estimate differences between the groups and time points. For correlation between lymph vessels, blood vessels, and GVHD, the Pearson coefficient was calculated. Survival was estimated using the Kaplan–Meier method and log-rank test. All statistical analyses were performed using SPSS (IBM, vs. 25). A *p*-value ≤ 0.05 was considered statistically significant by general convention.

## Figures and Tables

**Table 1:** RT-PCR primer (F = forward primer, R = reverse primer, VEGF = vascular endothelial growth factor, HPRT = hypoxianthine-guanine phosphoribosyl-transferase).

mRNA	Sequence	Product Size (bp)	Annealing Temperature
HPRT	F: 5′-CTTGGATACAGGCCAGACTTTGTTG-3′ R: 5′-GATTCAACTTGCGCTCATCTTAGGC-3′	163	60 °C
VEGF-C	F: 5′-AGAACGTGTCCAAGAAATCAGC-3′ R: 5′-ATGTGGCCTTTTCCAATACG-3′	219	55 °C

## Supplementary Materials

The following are available online at https://www.mdpi.com/article/10.3390/ijms22126191/s1.

## Abbreviations

AB	antibody
B6; 129s	mouse strains (B6 = C57/Bl6; 129s = 129S2/SvPasCrl)
BMC	bone marrow cells
BMT	bone marrow transplantation
CD	cluster of differentiation
ELG	extra-orbital lacrimal gland
FMO	fluorescence-minus-one
GVHD	graft-versus-host-disease
HLA	human-leukocyte antigen
IHC	immunohistochemistry
IV	intravenous injection
IP	intraperitoneal injection
MHC	major histocompatibility complex
miHA	minor histocompatibility antigen
PBS	phosphate-buffered solution
RT-PCR	real-time polymerase chain reaction
TBI	total body irradiation

## References

[1] Munir S.Z., Aylward J. (2017). A Review of Ocular Graft-Versus-Host Disease. Optom. Vis. Sci..

[2] Westeneng A.C., Hettinga Y., Lokhorst H., Verdonck L., van Dorp S., Rothova A. (2010). Ocular graft-versus-host disease after allogeneic stem cell transplantation. Cornea.

[3] Ogawa Y., Kim S.K., Dana R., Clayton J., Jain S., Rosenblatt M.I., Perez V.L., Shikari H., Riemens A., Tsubota K. (2013). International Chronic Ocular Graft-vs-Host-Disease (GVHD) Consensus Group: Proposed diagnostic criteria for chronic GVHD (Part I). Sci. Rep..

[4] Appenteng Osae E., Steven P. (2021). Meibomian Gland Dysfunction in Ocular Graft vs. Host Disease: A Need for Pre-Clinal Models and Deeper Insights. Int. J. Mol. Sci..

[5] Zeiser R., Blazar B.R. (2016). Preclinical models of acute and chronic graft-versus-host disease: How predictive are they for a successful clinical translation?. Blood.

[6] Zhang C., Todorov I., Zhang Z., Liu Y., Kandeel F., Forman S., Strober S., Zeng D. (2006). Donor CD4+ T and B cells in transplants induce chronic graft-versus-host disease with autoimmune manifestations. Blood.

[7] Hulsdunker J., Zeiser R. (2015). Insights into the pathogenesis of GvHD: What mice can teach us about man. Tissue Antigens.

[8] Boieri M., Shah P., Dressel R., Inngjerdingen M. (2016). The Role of Animal Models in the Study of Hematopoietic Stem Cell Transplantation and GvHD: A Historical Overview. Front. Immunol..

[9] Edinger M., Hoffmann P., Ermann J., Drago K., Fathman C.G., Strober S., Negrin R.S. (2003). CD4+CD25+ regulatory T cells preserve graft-versus-tumor activity while inhibiting graft-versus-host disease after bone marrow transplantation. Nat. Med..

[10] Taylor P.A., Lees C.J., Blazar B.R. (2002). The infusion of ex vivo activated and expanded CD4(+)CD25(+) immune regulatory cells inhibits graft-versus-host disease lethality. Blood.

[11] Mertlitz S., Shi Y., Kalupa M., Grotzinger C., Mengwasser J., Riesner K., Cordes S., Elezkurtaj S., Penack O. (2017). Lymphangiogenesis is a feature of acute GVHD, and VEGFR-3 inhibition protects against experimental GVHD. Blood.

[12] Penack O., Henke E., Suh D., King C.G., Smith O.M., Na I.K., Holland A.M., Ghosh A., Lu S.X., Jenq R.R. (2010). Inhibition of neovascularization to simultaneously ameliorate graft-vs-host disease and decrease tumor growth. J. Natl. Cancer Inst..

[13] Schroeder M.A., DiPersio J.F. (2011). Mouse models of graft-versus-host disease: Advances and limitations. Dis. Model. Mech..

[14] Passweg J.R., Baldomero H., Chabannon C., Basak G.W., de la Camara R., Corbacioglu S., Dolstra H., Duarte R., Glass B., Greco R. (2021). Hematopoietic cell transplantation and cellular therapy survey of the EBMT: Monitoring of activities and trends over 30 years. Bone Marrow Transplant..

[15] Penack O., Marchetti M., Ruutu T., Aljurf M., Bacigalupo A., Bonifazi F., Ciceri F., Cornelissen J., Malladi R., Duarte R.F. (2020). Prophylaxis and management of graft versus host disease after stem-cell transplantation for haematological malignancies: Updated consensus recommendations of the European Society for Blood and Marrow Transplantation. Lancet Haematol..

[16] Holtan S.G., Versluis J., Weisdorf D.J., Cornelissen J.J. (2021). Optimizing Donor Choice and GVHD Prophylaxis in Allogeneic Hematopoietic Cell Transplantation. J. Clin. Oncol..

[17] Hassan A.S., Clouthier S.G., Ferrara J.L., Stepan A., Mian S.I., Ahmad A.Z., Elner V.M. (2005). Lacrimal gland involvement in graft-versus-host disease: A murine model. Invest. Ophthalmol. Vis. Sci..

[18] Perez R.L., Perez-Simon J.A., Caballero-Velazquez T., Flores T., Carrancio S., Herrero C., Blanco B., Gutierrez-Cosio S., Canete-Campos C., Cruz Gonzalez F. (2011). Limbus damage in ocular graft-versus-host disease. Biol. Blood Marrow Transpl..

[19] Herretes S., Ross D.B., Duffort S., Barreras H., Yaohong T., Saeed A.M., Murillo J.C., Komanduri K.V., Levy R.B., Perez V.L. (2015). Recruitment of Donor T Cells to the Eyes During Ocular GVHD in Recipients of MHC-Matched Allogeneic Hematopoietic Stem Cell Transplants. Investig. Ophthalmol. Vis. Sci.

[20] He J., Yamane M., Shibata S., Fukui M., Shimizu E., Yano T., Mukai S., Kawakami Y., Li S., Tsubota K. (2018). Ocular Surface and Tear Film Characteristics in a Sclerodermatous Chronic Graft-Versus-Host Disease Mouse Model. Cornea.

[21] Perez V.L., Barsam A., Duffort S., Urbieta M., Barreras H., Lightbourn C., Komanduri K.V., Levy R.B. (2016). Novel Scoring Criteria for the Evaluation of Ocular Graft-versus-Host Disease in a Preclinical Allogeneic Hematopoietic Stem Cell Transplantation Animal Model. Biol. Blood Marrow Transpl..

[22] Muller A.M.S., Min D., Wernig G., Levy R.B., Perez V.L., Herretes S., Florek M., Burnett C., Weinberg K., Shizuru J.A. (2019). Modeling Chronic Graft-versus-Host Disease in MHC-Matched Mouse Strains: Genetics, Graft Composition, and Tissue Targets. Biol. Blood Marrow Transpl..

[23] Yamane M., Sato S., Shimizu E., Shibata S., Hayano M., Yaguchi T., Kamijuku H., Ogawa M., Suzuki T., Mukai S. (2020). Senescence-associated secretory phenotype promotes chronic ocular graft-vs-host disease in mice and humans. FASEB J..

[24] Shamloo K., Barbarino A., Alfuraih S., Sharma A. (2019). Graft Versus Host Disease-Associated Dry Eye: Role of Ocular Surface Mucins and the Effect of Rebamipide, a Mucin Secretagogue. Investig. Ophthalmol. Vis. Sci..

[25] Riesner K., Kalupa M., Shi Y., Elezkurtaj S., Penack O. (2016). A preclinical acute GVHD mouse model based on chemotherapy conditioning and MHC-matched transplantation. Bone Marrow Transpl..

[26] Riesner K., Shi Y., Jacobi A., Krater M., Kalupa M., McGearey A., Mertlitz S., Cordes S., Schrezenmeier J.F., Mengwasser J. (2017). Initiation of acute graft-versus-host disease by angiogenesis. Blood.

[27] Chennakesavalu M., Somala S.R.R., Dommaraju S.R., Peesapati M.P., Guo K., Rosenblatt M.I., Chang J.H., Azar D.T. (2021). Corneal Lymphangiogenesis as a Potential Target in Dry Eye Disease—A Systematic Review. Surv. Ophthalmol..

[28] Goyal S., Chauhan S.K., El Annan J., Nallasamy N., Zhang Q., Dana R. (2010). Evidence of corneal lymphangiogenesis in dry eye disease: A potential link to adaptive immunity?. Arch. Ophthalmol..

[29] Ji Y.W., Lee J.L., Kang H.G., Gu N., Byun H., Yeo A., Noh H., Kim S., Choi E.Y., Song J.S. (2018). Corneal lymphangiogenesis facilitates ocular surface inflammation and cell trafficking in dry eye disease. Ocul. Surf..

[30] Dietrich-Ntoukas T., Cursiefen C., Westekemper H., Eberwein P., Reinhard T., Bertz H., Nepp J., Lawitschka A., Heiligenhaus A., Seitz B. (2012). Diagnosis and treatment of ocular chronic graft-versus-host disease: Report from the German-Austrian-Swiss Consensus Conference on Clinical Practice in chronic GVHD. Cornea.

[31] Kitko C.L., Pidala J., Schoemans H.M., Lawitschka A., Flowers M.E., Cowen E.W., Tkaczyk E., Farhadfar N., Jain S., Stevens P. (2021). National Institutes of Health Consensus Development Project on Criteria for Clinical Trials in Chronic Graft-versus-Host Disease: IIa. The 2020 Clinical Implementation and Early Diagnosis Working Group Report. Transpl. Cell Ther..

[32] Bock F., Maruyama K., Regenfuss B., Hos D., Steven P., Heindl L.M., Cursiefen C. (2013). Novel anti(lymph)angiogenic treatment strategies for corneal and ocular surface diseases. Prog. Retin. Eye Res..

[33] Park P.J., Chang M., Garg N., Zhu J., Chang J.H., Shukla D. (2015). Corneal lymphangiogenesis in herpetic stromal keratitis. Surv. Ophthalmol..

[34] Hos D., Matthaei M., Bock F., Maruyama K., Notara M., Clahsen T., Hou Y., Le V.N.H., Salabarria A.C., Horstmann J. (2019). Immune reactions after modern lamellar (DALK, DSAEK, DMEK) versus conventional penetrating corneal transplantation. Prog. Retin. Eye Res..

[35] Cursiefen C. (2007). Immune privilege and angiogenic privilege of the cornea. Chem. Immunol. Allergy..

[36] Yang J.F., Walia A., Huang Y.H., Han K.Y., Rosenblatt M.I., Azar D.T., Chang J.H. (2016). Understanding lymphangiogenesis in knockout models, the cornea, and ocular diseases for the development of therapeutic interventions. Surv. Ophthalmol..

[37] Goyal S., Chauhan S.K., Dana R. (2012). Blockade of prolymphangiogenic vascular endothelial growth factor C in dry eye disease. Arch. Ophthalmol..

[38] Penack O., Socie G., van den Brink M.R. (2011). The importance of neovascularization and its inhibition for allogeneic hematopoietic stem cell transplantation. Blood.

[39] Regenfuss B., Bock F., Cursiefen C. (2012). Corneal angiogenesis and lymphangiogenesis. Curr. Opin. Allergy. Clin. Immunol..

[40] Regenfuss B., Onderka J., Bock F., Hos D., Maruyama K., Cursiefen C. (2010). Genetic heterogeneity of lymphangiogenesis in different mouse strains. Am. J. Pathol..

[41] Horstmann J., Schulz-Hildebrandt H., Bock F., Siebelmann S., Lankenau E., Huttmann G., Steven P., Cursiefen C. (2017). Label-Free In Vivo Imaging of Corneal Lymphatic Vessels Using Microscopic Optical Coherence Tomography. Investig. Ophthalmol. Vis. Sci..

[42] Le V.N.H., Hou Y., Horstmann J., Bock F., Cursiefen C. (2018). Novel Method to Detect Corneal Lymphatic Vessels In Vivo by Intrastromal Injection of Fluorescein. Cornea.

[43] Gehlsen U., Braun T., Notara M., Krosser S., Steven P. (2017). A semifluorinated alkane (F4H5) as novel carrier for cyclosporine A: A promising therapeutic and prophylactic option for topical treatment of dry eye. Graefes Arch. Clin. Exp. Ophthalmol..

[44] Frangi A., Niessen W.J., Vincken K.L., Viergever M.A., Wells W., Colchester A., Delp S. (2006). Multiscale vessel enhancement filtering. Medical Image Computing and Computer-Assisted Intervention.

[45] Otsu N. (1979). A threshold selection method from gray-level histograms. IEEE Trans. Syst. Man Cybern..

