# Supplementary material for: Ocular Graft-versus-Host Disease in a Chemotherapy-Based Minor-Mismatch Mouse Model Features Corneal (Lymph-) Angiogenesis
Source: Int J Mol Sci. 2021 Jun 8;22(12):6191. doi: 10.3390/ijms22126191 (PMC8228997; doi:10.3390/ijms22126191)
Supplement: Supplementary file 1 [file ijms-22-06191-s001.zip › ijms-1249475-supplementary.pdf]

Supplementary Material

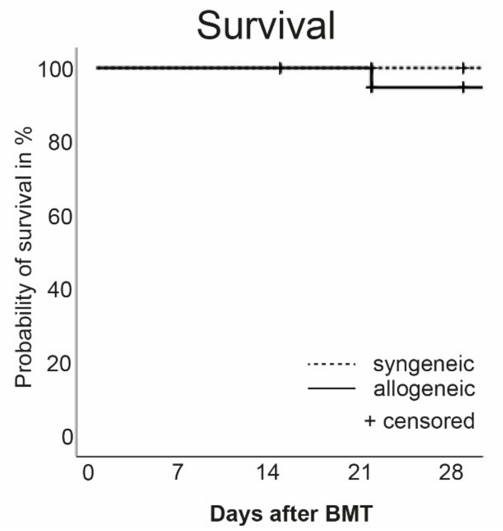

Scheme S1. Overall survival of mice after syngeneic or allogeneic BMT until termination of the experiments at day 28. Mice that were sacrificed on purpose before day 28 to be further analyzed were censored. No differences in the probability of survival between the groups were observed (log-rank test: p=0.4).

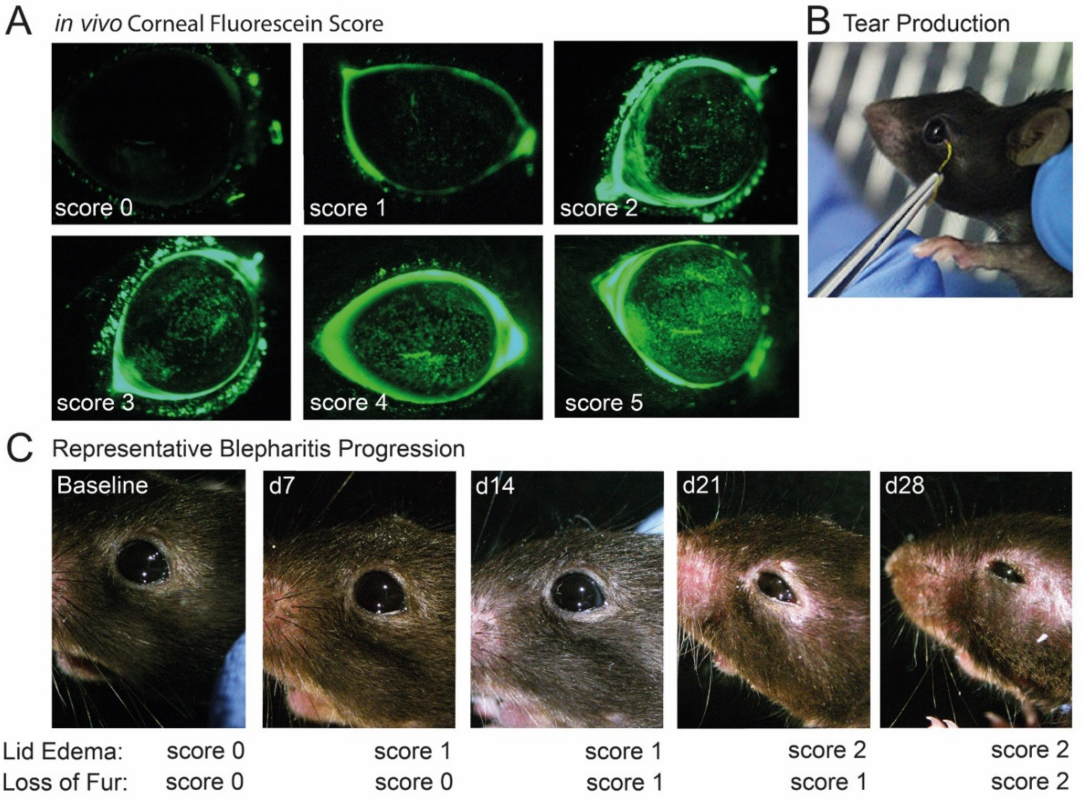

Scheme S2. Representative images of **A)** corneal fluorescein score, **B)** assessment of tear production using a phenol red thread and **C)** typical progression of blepharitis in experimental GVHD, following the same allogeneic transplanted mouse from baseline to day 28 after BMT. Lid edema/swelling and periocular loss of fur were scored individually from 0-2 and summated to the final blepharitis score.

Scheme S3. Antibodies.

Antibody-*conjugate* (clone)	Target	Manufacturer (catalog no.)
**Antibodies used for Flow Cytometry**		
anti-mouse-CD4 ***FITC*** (RM 4-5)	CD4+ T cells	Biolegend (#100509)
anti-mouse-CD8a ***APC*** (53-6.7)	CD8+ T cells	Biolegend (#100711)
anti-mouse-CD3 ***BV421*** (17A2)	pan T cells	Biolegend (#100227)
anti-mouse-CD45 ***PE*** (PC61)	pan leucocytes	Biolegend (#102007)
Fixable Viability Dye ***eFluor*™*780***	dead cells	Invitrogen (#65-0865-14)
anti-mouse-Ly9.1 ***PE*** (30C7)	CD229+ thymocytes	BD Pharmingen (#560201)
anti-mouse-H2ƙb ***FITC*** (AF6-88.5)	MHC I ƙb	BioLegend (#116505)
**Antibodies used for IHC**		
rat-anti-mouse-CD4 (H129.19)	CD4+ T cells	BD Bioscience (#553647)
rat-anti-mouse-CD8a (53-6.7)	CD8+ T cells	Invitrogen (#14-0081-82)
rabbit-anti mouse-LYVE-1	Lymphatics	Abcam (#ab14917)
rat-anti-mouse-CD31	Blood vessels	BD Pharmingen (#558738)
goat-anti-rat-Alexa Fluor™ 555	Secondary AB	Invitrogen (#A21434)
goat-anti-rabbit-***Cy3***™	Secondary AB	Jackson ImmunoResearch (#111-165-045)
